# Protocol for generating and characterizing Matrigel-free prostate cancer patient-derived organoids

**DOI:** 10.1016/j.xpro.2026.104486

**Published:** 2026-04-07

**Authors:** Robin Dolgos, Romuald Parmentier, Jing Wang, Arnoud J. Templeton, Kirsten D. Mertz, Heike Pueschel, Helge Seifert, Ashkan Mortezavi, Tatjana Vlajnic, Cyrill A. Rentsch, Lukas Bubendorf, Clémentine Le Magnen

**Affiliations:** 1 Institute of Medical Genetics and Pathology, University Hospital Basel, Basel, Switzerland; 2 Department of Urology, University Hospital Basel, Basel, Switzerland; 3 Department of Biomedicine, University of Basel, University Hospital Basel, Basel, Switzerland; 4 Division of Medical Oncology, St. Claraspital, Basel, and Faculty of Medicine, University of Basel, Basel, Switzerland

**Keywords:** Bioinformatics, Cancer, Cell Biology, Molecular Biology, Organoids

## Abstract

Here, we present a protocol for the generation of prostate cancer patient-derived organoids (PCa PDOs) in Matrigel-free conditions. We describe steps for sample selection, pathological resection, and tissue dissociation. We then detail procedures for the generation, maintenance, and characterization of the PCa PDOs. Finally, we provide guidance for the processing of PDOs for single-cell RNA sequencing (scRNA-seq) analysis and using the prostate PDO single-cell atlas (PPScA) for both novice and advanced bioinformatic users.

For complete details on the use and execution of this protocol, please refer to Dolgos et al.^1^

## Highlights

• Optimized dissociation workflow for prostate cancer (PCa) tumors
• Protocol for generating Matrigel-free PCa patient-derived organoids
• Procedures to characterize organoids when working with limited organoid numbers
• Preparation of organoids for scRNA-seq and tutorial for PCa PDO single-cell atlas

## Abstract (editor highlights)

Publisher’s note: Undertaking any experimental protocol requires adherence to local institutional guidelines for laboratory safety and ethics.

## Graphical abstract

**Figure:**
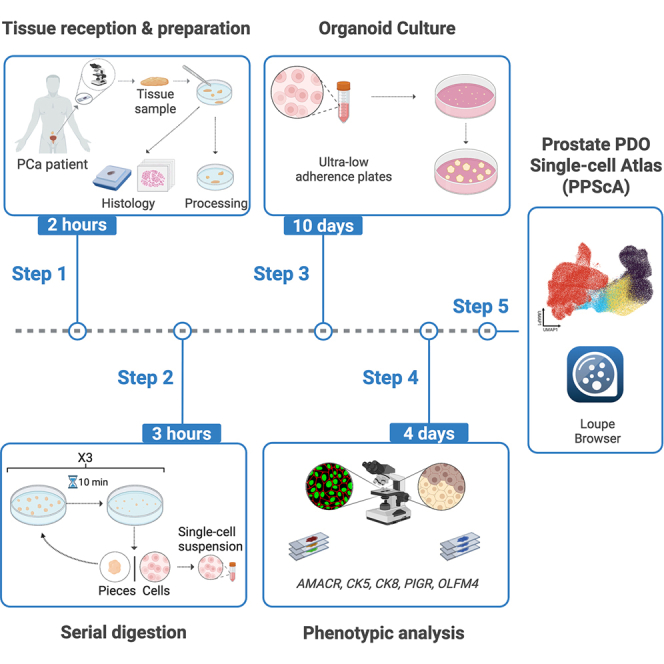

## Before you begin

See the full list of reagents in the key resources table. Perform all centrifugation steps of cells and organoids at 350 g for 5 min in spinning buckets.

As this protocol includes human tissues, verify that you comply with applicable local, institutional and national regulations. Ensure that required approvals, consent documentation, and ethical clearances are in place prior to initiating the work. In the context of our specific study, informed consent is obtained from each enrolled patient before sample collection, under an approval by the Ethics Committee of Northwestern and Central Switzerland (EKBB/EKNZ 37/13).

While this protocol describes the steps for Matrigel-free organoid culture conditions, a subset of advanced prostate cancer samples may grow successfully and expand in classical Matrigel-based conditions (as pointed in our study^1^). Particularly in the case of metastasis samples, we advise to grow the samples in both Matrigel-free and Matrigel-based conditions, whenever possible. For the latter, we refer the readers to seminal works reporting Matrigel-based culture for PCa PDOs.^2^^,^^3^^,^^4^^,^^5^^,^^6^ All the subsequent steps for organoid characterization can be applied for both culture condition systems.

### Innovation

Traditional prostate cancer patient-derived organoid culture relies on an Engelbreth–Holm–Swarm (EHS) mouse–tumor–derived extracellular matrix preparation (Matrigel). Although this approach has been effective for many cancer types, Matrigel-based culture often associates with low take-rates and overgrowth of benign epithelial cells in the context of PCa. By optimizing tumor digestion conditions and eliminating the extracellular matrix, our Matrigel-free protocol improves both the take-rate and the proportion of tumor cells within PCa PDO cultures, as demonstrated in our recent publication.^1^ Using this method, we successfully maintained PCa PDOs for up to five passages, during which they retained higher degree of phenotypic and transcriptomic similarity with their matched parental tumors. By generating and sharing our user-friendly Prostate PDO single-cell Atlas (PPScA), we allow researchers to interrogate transcriptomic profiles of organoids at single-cell level and provide a dataset that can drive further optimization of culture conditions by other groups.

## Key resources table

**Table:** 

REAGENT or RESOURCE	SOURCE	IDENTIFIER
**Antibodies**		

Androgen Receptor Rabbit mAb primary antibody	Abcam	ab133273
Cytokeratin 5 Chicken pAb primary antibody	Biolegend	905901
Cytokeratin 8 Mouse mAb primary antibody	Biolegend	904801
AMACR Rabbit mAb primary antibody	Cell Signaling	29256
OLFM4 Rabbit mAb primary antibody	Cell Signaling	14369
MUC5AC Mouse mAb primary antibody	Invitrogen	MA5-12178
Goat anti-Chicken IgY Alexa Fluor® 555 secondary antibody	Invitrogen	A-21437
Goat anti-Chicken IgY Alexa Fluor® 488 secondary antibody	Invitrogen	A-11039
Goat anti-Rabbit IgG Alexa Fluor® 555 secondary antibody	Invitrogen	A-21428
Goat anti-Mouse IgG Alexa Fluor® 647 secondary antibody	Invitrogen	A-21237

**Chemicals, peptides, and recombinant proteins**		

B27	Life Technologies	17504044
Nicotinamide	Sigma-Aldrich	N0636-100g
*N*-Acetyl-l-cysteine	Sigma-Aldrich	A9165-25g
A83-01	Tocris	2939
FGF-10	Peprotech	100-26-25UG
FGF-2	Peprotech	100-18B-50UG
EGF	Peprotech	AF-100-15-100UG
Noggin	Peprotech	120-10C-50UG
Rspondin 1	R&D	4645-RS
Prostaglandin E2	Tocris	2296
SB202190	Sigma-Aldrich	S7076-5MG
Y-27632 dihydrochloride	Abmole Bioscience	M1817
(Dihydro)testosterone (5α-androstan-17β-ol–3-one)	Sigma-Aldrich	10300-5G-F
Growth Factor Reduced Matrigel	Corning	354230
TrypLE Express Enzyme	Gibco	12605010
GlutaMAX Supplement	Gibco	35050061
HEPES	Gibco	15630080
Advanced DMEM/F12	Gibco	12634010
Red Blood Cell Lysis Buffer	Roche	11814389001
Dead Cell Removal Kit	Miltenyi Biotech	130090101
Primocin	InvivoGen	ant-pm-1
Penicillin-Streptomycin	Sigma-Aldrich	P4333-100ML
Collagenase Type 4	Worthington	LS004189
DISPASE	Gibco	17105041
Normal Goat Serum	Vector Laboratories	S-1000-20
Tween	Sigma-Aldrich	P9416-100ML
Q Retrieval Low pH 6.0 (50×) Citrate Buffer	quartett	AR-001-0120
Histogel	epredia	HG-4000-012
Superforst Plus Adhesion Microscope Slides	epredia	J1840AMNZ
Cover Slips	epredia	24 × 50 mm #1
Fluoromount Aqueous Mounting Medium	Sigma-Aldrich	F4680-25ML
A-PAP PEN Mini	Daido Sangyo	DAI-PAP-R
Hypothermosol FRS	Sigma-Aldrich	H4416
Bovine Serum Albumin	Sigma-Aldrich	A3773-50G
DNAse I	Sigma-Aldrich	11284932001

## Step-by-step method details

### Sample selection

**Timing: 3 h**

1. Select sample in close collaboration with involved study nurses, surgeons, oncologists, and pathologists.
  ***Note:*** All types of prostate cancer surgical samples can be used with various success rates. In particular, our group has had success with radical prostatectomies (RPs), transurethral resection of the prostate (TURPs), prostate biopsies, as well as metastasis biopsies and resections.
  a. To generate organoids with the highest tumor content, select samples according to the following criteria:
    i. A minimum ISUP Grade Group of 3.
    ii. In case previous biopsies were performed: high proportion of positive biopsies (e.g., >50% overall or in targeted biopsies from PIRADS lesion), preferably with high tumor content.
    iii. Samples with known genomic alterations such as PTEN alterations or ERG translocations are preferred. They allow identification of tumor cells based on biomarker positivity in matched organoids.
    iv. High proliferation (10–15% KI67 positive tumor cells).
    v. Additionally, prioritize samples with high PSA, suspicion of metastasis, intraductal or cribriform patterns, and large lesions visible by imaging.

### Sample transport and pathological evaluation

**Timing: 1 h**

2. Optimize the sample route so that the sample reaches the pathologist as fast as possible (ideally within 1 hour).

**Figure 1:**
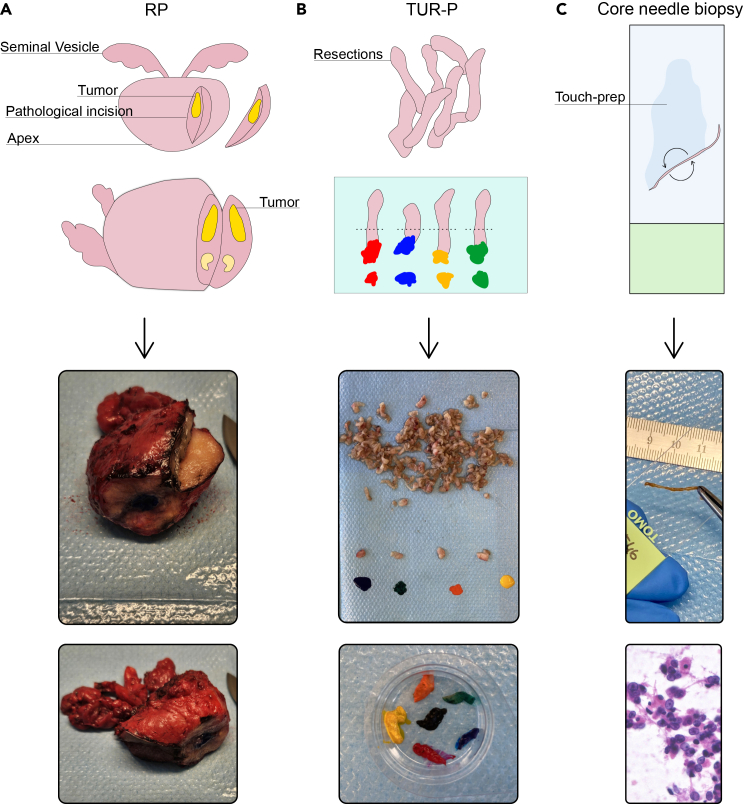
Pathological evaluation and sample selection for RP, TURP and prostate biopsies (A) Sample processing for radical prostatectomies. (B) Sample processing for transurethral resections of the prostate. (C) Sample processing for needle core biopsies (prostate or metastasis). The H&E microscopy image shows prostate tumor cells detected in a touch-prep.

**CRITICAL:** Always transport the sample in a physiological solution (0.9% NaCl, PBS or ADMEM).

***Optional:*** If transport exceeds 1 h or the sampling is done overnight, preserve sample at 4°C in Hypothermosol preservation buffer (key resources table).

***Note:*** Pathological evaluation of all clinically relevant parameters has priority.

3. If tissue is left over, identify and resect a piece of tissue with the highest possible tumor content for organoid experiments.
  ***Note:*** When available, imaging information associated with the patient may be used to guide pathological resection.
  a. RP specimens:
    i. If tumor tissue is not evident by macroscopy in radical prostatectomy specimens, perform frozen section analysis to confirm cancer and maximize tumor proportion in the specimen (Figure 1A).
    ii. Guide the collection of material by localization based on pre-operative MRI.
  b. TURP specimens:
    i. Cut 5–6 macroscopically suspicious tissue fragments in half.
    ii. Ink each of these with different colors for subsequent frozen section analysis.
      ***Note:*** The different colors allow for frozen section analysis of all specimens at a time (Figure 1B).
    iii. Moisten the un-inked half of the matched tissue fragments to avoid drying while waiting for the frozen section.
    iv. Select the tumor-containing tissue fragments based on frozen section for further processing.
  c. Core needle biopsies:
    i. Urologists should collect an extra 1–3 core needle biopsies for the purpose of generating organoids and label the transport container accordingly.
      ***Note:*** Frozen section of small biopsies should be avoided to prevent wasting the tissue.
    ii. Use cytological touch preparations to ascertain the presence of tumor cells (Figure 1C).
  d. Metastasis biopsies/resections:
    i. Same principles as for radical prostatectomy, TURP specimens or core needle biopsies, depending on the size of the tissue specimens.
    ii. In case of biopsies, perform cytological touch preparations for tumor cell confirmation.
      ***Note:*** The minimum tissue amount required to initiate a culture is approximatively 1 prostate biopsy, or 2.5 mm^3^ pieces of RP, TURP, or metastasis resections.
4. Once prepared and selected for further processing, keep the sample in solution and transport to BSL2 laminar flow hood.

### Digestion mix preparation

**Timing: 15 min**

***Note:*** Digestion mix should be prepared fresh each time. To streamline the process, prepare batches of 5 non-resuspended digestion mixes in 50 mL Falcon tube and keep them at 4°C. This is only possible if the enzymes available in your region are compatible with long-term storage at 4°C.

5. To prepare digestion mix, weigh and combine the following powders in a 50 mL falcon tube:
  a. 19U of Dispase, 5000U of Collagenase IV.
6. Resuspend in 10 mL of aDMEM and filter with a 0.22 μm filter.
7. Add 10 μg DNase I and 10 μL of 1 mM of Y-27632.

### Single-cell suspension generation by serial digestion

**Timing: 1–4 h**

8. Place the sample in a 75 mm petri dish.
  a. Use a scalpel and tweezers to cut the sample in small 5 mm pieces.
9. Transfer a piece to a 1.5 mL Eppendorf tube.
  a. Add 1 mL of 4% Paraformaldehyde for histology.
  b. Store overnight (14**–**20 h) at 4°C for complete fixation.
10. Place the remaining pieces of tissue in another 1.5 mL Eppendorf.
  a. Cover with digestion mix (100**–**1500 μL).

***Note:*** Add the minimum amount of volume to cover the pieces as this will facilitate the mincing step.

11. Use a Westcott surgical scissor to mince the tissue pieces as small as possible (for 5**–**10 min).
  a. Transfer the solution to a new 75 mm petri dish and add 10 mL of digestion mix.
    ***Note:*** A minimum of 10 mL is necessary to have sufficient volume in a 75 mm petri dish, even with smaller tissue fragments.
  b. Place on a rotating platform (20–30RPM depending on inclination) at 37°C in an incubator for 10 min.
12. After performing 5–10 resuspensions by pipetting, transfer the solution to a 15 mL Falcon tube.
  a. Carefully invert the falcon multiple times to mix the contents.
    i. Wait for the larger pieces to sink and create a pellet.
    ii. As soon as the largest pieces have sunk, pipet the supernatant and transfer to a new 15 mL falcon.
      ***Note:*** If needed, the digestion mix for the next step can be taken from the supernatant after centrifuging.
  b. Add 10 mL of digestion mix to the remaining chunks.
    i. Place back into the petri dish.
    ii. Place on a rotating platform (20–30RPM depending on inclination) at 37°C in an incubator for 10 min.
13. Centrifuge the falcon with the supernatant, resuspend in aDMEM and count the cells.
  a. Repeat this step 2–5 times depending on sample size, and number of cells released at each serial passage.
    i. Keep the cell suspensions separate, in case you want to exclude certain rounds of digestion due to low viability.
14. Pool cells you wish to include in the culture (you may choose to set a threshold of viability >5%), and filter at 50 μm before centrifuging.
  a. Resuspend the cell pellet in aDMEM (100–1000 μL, depending on the size of the pellet) and proceed to cell count.

**Figure 2:**
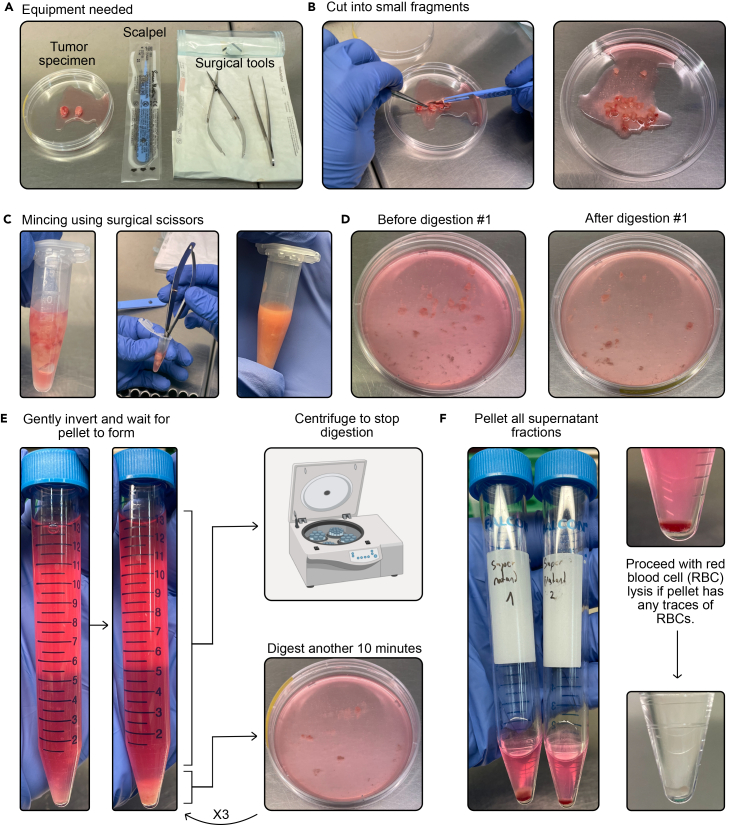
Major dissociation steps. Example of two lymph node metastases obtained from one patient (P25–45) (A) Surgical tools needed to cut and mince the surgical samples. (B) Surgical sample should be cut in ∼5 mm pieces. One piece should be kept for histological analysis, and another snap frozen for DNA/RNA extraction. (C) Small pieces should be transferred to an Eppendorf and covered with digestion mix, before being minced using micro-scissors for ∼5 min. (D) After 10 min (digestion #1), large pieces will remain. (E) Transfer the mixture to a 15 mL falcon tube, invert, and wait for the pellet to form before transferring the supernatant to another Falcon. Stop the supernatant #1s digestion. Resuspend the pellet in digestion mix and digest for another 10 min. Repeat the process 2 additional times. (F) Pellet all supernatant fractions and if necessary, proceed with RBC.

***Optional:*** Red Blood Cell Lysis. If the final cell pellet contains traces of red blood cells (Figure 2F), proceed with red blood cell lysis according to manufacturer recommendations (link).

### Organoid medium

**Timing: 30 min**

15. Keep the organoid medium for a maximum of 7 days at 4°C.
  a. Add DHT and Y-27632 fresh each time.
  b. Prepare a “base medium” composed of 500 mL AdDMEM-F12, 5 mL Glutamax, 5 mL Hepes, 5 mL Pen/Strep, and 100 μg/mL Primocin.
  c. Use this “base medium” to prepare complete medium aliquots (Table 1).

**Table 1:** Organoid medium composition

Component	Concentration/Volume
adMEM F12	500 mL
Glutamax	1%
Hepes	1%
Pen/Strep	1%
Primocin	100 μg/mL
B27	1×
Nicotinamide	10 mM
N-Acetyl-L-cysteine	1.25 mM
A83-01	500 nM
FGF-10	5 ng/mL
FGF-2	5 ng/mL
EGF	5 ng/mL
Prostaglandin E2	1 μM
  d. The organoid medium composition is adapted from seminal works in the field.^2^

### PDO generation

**Timing: 3–7 days**

16. For PDO generation, resuspend the cells in complete organoid medium.
  a. Adhere to the following guidelines to choose the appropriate ultra-low adherence plate size (Table 2).

**Table 2:** Seeding density for organoid generation in ultra-low adherence plates

Cell count	Well to use	Volume of plate
5,000–20,000	1 well of 96-well plate	50–100 μL
20,000–50,000	1 well of 48-well plate	500 μL
50,000–200,000	1 well of 24-well plate	1000 μL
200,000–500,000	1 well of 6-well plate	2000 μL

### Organoid medium renewal

**Timing: 1 h**

17. Organoid medium should be changed twice a week.
  a. To avoid losing cells and to account for the general low cell numbers associated to PCa samples, avoid complete medium removal by centrifugation.
    i. Instead, change 75% of medium volume.
  b. Gently tilt the plate and wait 30 seconds to allow for the PDOs to move to the lowest part of the plate.
  c. Gently aspirate 75% of the organoid medium by placing the pipette tip at the highest point of the volume.

***Note:*** When preparing the complete organoid medium, consider the total volume of the well when adding Y-27632 and DHT (consider 2 mL when you add 1.5 mL of medium).

### Organoid passaging

**Timing: 1–2 h**

***Note:*** Organoids should be passaged every 7**–**10 days (Figure 3).

18. To collect the organoids while leaving the dead cells in the well, transfer the entire volume of the well in a 15 mL Falcon tube.
  a. After gently inversing the falcon 2**–**3 times to mix contents, allow for the organoids to form a pellet (can take up to 5 min).
  b. Once the pellet has formed, check that no organoids are remaining in the supernatant using the brightfield microscope.
    i. Remove the supernatant containing the dead cells.

**Figure 3:**
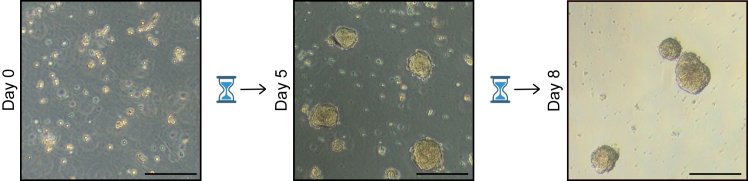
P25–45 patient-derived organoids across 8 days of culture (Passage 0) Scale bars represent 200 μm.
19. Resuspend the pellet in 1 mL of pre-warmed TrypLE containing 1 mM of Y-27632.
  a. Transfer to a 37°C water bath.
20. Every 2 min, gently pipet to facilitate digestion and check the state of organoids under the brightfield microscope.
  a. When 90% of organoids are dissociated, stop digestion by adding 10 mL of adMEM ++++ and centrifuge.

**Figure 4:**
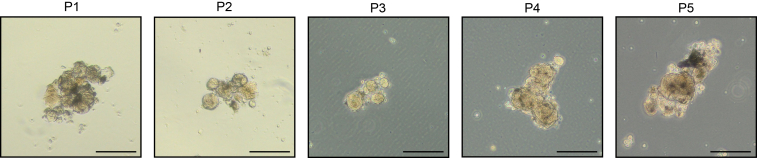
Example of organoid passages for sample P23-20 Scale bars represent 200 μm.

***Note:*** We were able to keep multiple organoid cultures up to passage 5, as shown in Figure 4.

### Embedding of organoids in Histogel

**Timing: 2–14 h**

21. Fix the organoids in 4% formalin for 1 h at RT or overnight (14–20 h) at 4°C.

**Figure 5:**
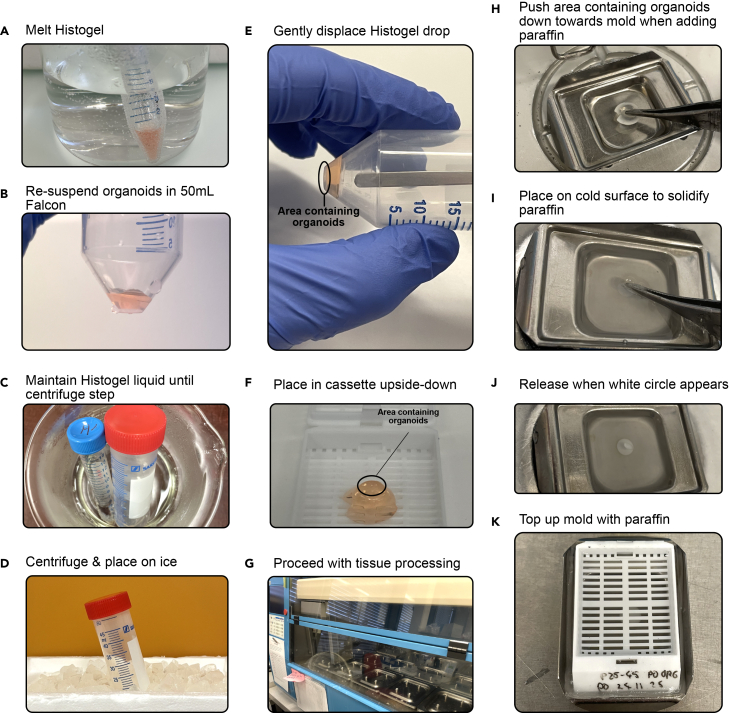
Preparation of organoid FFPE blocks for histology (A) Melt Histogel in beaker containing water. (B) Resuspend organoids in 300 μL of Histogel and transfer to a 50 mL Falcon. (C) Maintain Histogel liquid by keeping it in warm water until centrifuge step. (D) Quickly transfer to a centrifuge, and place on ice for 30 min minimum. (E) Gently displace Histogel drop with a round and thin metal spatula. (F) Transfer the Histogel drop to a tissue processing cassette, upside-down to ensure organoids are not damaged or lost. (G) Proceed with tissue processing as detailed in Table 3. (H) Place the Histogel drop face down (i.e., smaller side with organoids facing down) and apply some pressure with metal tweezers while adding paraffin. (I) While maintaining pressure, move mold to a cold surface to solidify paraffin. (J) Release pressure and remove pliers when the Histogel drop solidifies in the paraffin, which can be identified by the formation of a white circle. (K) Top up mold with paraffin and add cassette on top.
22. Warm up the Histogel until it undergoes liquefaction.
  a. Keep the Histogel tube in a larger beaker with hot water to keep it liquid during the remaining steps (Figure 5C).
    **CRITICAL:** If using a microwave, keep the lid unscrewed and check every 30 s to minimize explosion hazards.
  b. Place one 50 mL Falcon tube per sample you wish to process in the hot water beaker/container to pre-warm it.
23. Centrifuge your fixed organoids and remove supernatant.
  a. Using a cut pipette-tip, resuspend the organoids with Histogel.
    i. Quickly transfer to a pre-warmed 50 mL Falcon.

**CRITICAL:** Ensure that no air bubbles are introduced when resuspending organoids in histogel.

24. Place back all 50 mL Falcon tubes containing the organoids in the warm water until you have finished processing them all.
25. Quickly transfer the Falcon tubes to a centrifuge, and spin for 5 min at 350 g for the organoids to assemble towards the tip of the Falcon.
26. Place the falcon tubes on ice for 30 min minimum.
  a. Remove the solidified pellet with a small spatula by carefully pushing down on one side.
  b. Transfer the solidified pellet into an embedding cassette and proceed with tissue processing.

### Paraffin embedding

**Timing: 12–14 h**

27. Place the embedding cassettes in the tissue processor buckets, and set-up the machine with the steps described in Table 3.

**Table 3:** Tissue processing steps

Number	Bath	Time	Temperature
1	Alcohol 70%	1 h	37°C
2	Alcohol 70%	1 h	37°C
3	Alcohol 70%	1 h	37°C
4	Alcohol 80%	1 h	37°C
5	Alcohol 96%	1 h	37°C
6	Alcohol 96%	1 h	37°C
7	Alcohol 100%	1 h	37°C
8	Alcohol 100%	1 h	37°C
9	Xylene	1 h	37°C
10	Xylene	1 h	37°C
11	Paraffin	1 h	60°C
12	Paraffin	1 h	60°C
13	Paraffin	1 h	60°C

**CRITICAL:** Heated organic solvents are highly volatile, and should be kept under separate ventilation systems.

28. Place cassette in 60°C drawer of embedding station.
  a. Remove Histogel structure from the cassette and transfer it to an embedding mold.
  b. Ensure that the smaller end of the Histogel structure containing the organoids is facing down against the mold (Figure 5H).
29. While carefully pressing down on the Histogel structure using forceps, proceed with embedding.

### Cutting of FFPE blocks containing organoids

**Timing: 1–4 h**

**CRITICAL:** To reach the FFPE block with the blade on the microtome, do not use the “trim” function, always stay on 3.5 μm increments.

30. Using a microtome, start cutting 3.5 μm slides until you reach the FFPE block.
31. As soon as the FFPE block is reached by the blade, visualize the Histogel drop on your block.
  a. As soon as even the slightest portion of the Histogel drop is being cut, transfer the slice to a slide and verify if organoids are present under a brightfield microscope.
  b. Repeat until organoids are captured.

***Note:*** In our experience, organoids are often found in the sides of the Histogel drop. Keep that in mind when cutting.

### Immunofluorescence

**Timing: 2 days**

32. Preparation of slides.
  a. Melt the paraffin for 30 min at 60°C.
  b. Remove excess paraffin in Xylene, 2 times 7.5 min.
  c. Soak in 100% EtOH, 2 times 3 min.
  d. Soak in 96% EtOH 2 min.
  e. Soak in 80% EtOH 2 min.
  f. Soak in 70% EtOH 2 min.
  g. Soak in dH2O 5 min.
33. Antigen retrieval.
  a. Dilute 1.5 mL of citrate buffer pH 6 (stock 50×) in 75 mL dH2O.
  b. Proceed with antigen retrieval program.
  c. Program: Normal pressure, 98°C for 15 min.
    i. Let cool down for 30 min at RT.
  d. Soak slides in dH2O 2 times 5 min.
  e. Soak slides in PBS pH 7.4 for 10 min on shaking platform.

***Note:*** You may need to optimize the antigen retrieval program for certain antibodies and antigens.

34. Permeabilization.
  a. Soak slides in PBS 0.2% Tween on shaking platform, 2 times 15 min.
  b. Wash slides in PBS pH 7.4, 2 times 5 min on shaking platform.
35. Blocking.
  a. Prepare slides (2 slides at a time) with hydrophobic pen by circling the sections.
  b. Block with 80 μL 10% goat serum (if secondary antibodies are produced in goat) in PBS for 1 hour at RT in humid chamber.
36. Primary antibody reaction.
  a. Flush the blocking solution on paper towel.
  b. Add 80 μL of primary antibody mix at adequate dilution in PBS 1% goat serum.
  c. Incubate overnight at 4°C in a humidified container.
37. Secondary antibody reaction.
  a. Wash slides in PBS pH 7.4 on a shaker, 3 times 5 min.
  b. Incubate 1 h in dark humid chamber at RT.
  c. Wash slides in PBS pH 7.4 on a shaker, 3 times 5 min.
38. Mounting the slides.
  a. Mount the slides with IF mounting medium.
    i. Remove all air bubbles with a pipette tip, by gently pressing on the cover slip.
    ii. Remove excess mountain medium by tilting the slides and gently touching the slides with a paper towel.
  b. Let the slides dry flat in a dry chamber for 2 h.
  c. Keep at 4°C in the dark until analysis.
    i. Slides can be imaged for up to 3 weeks.

**Figure 6:**
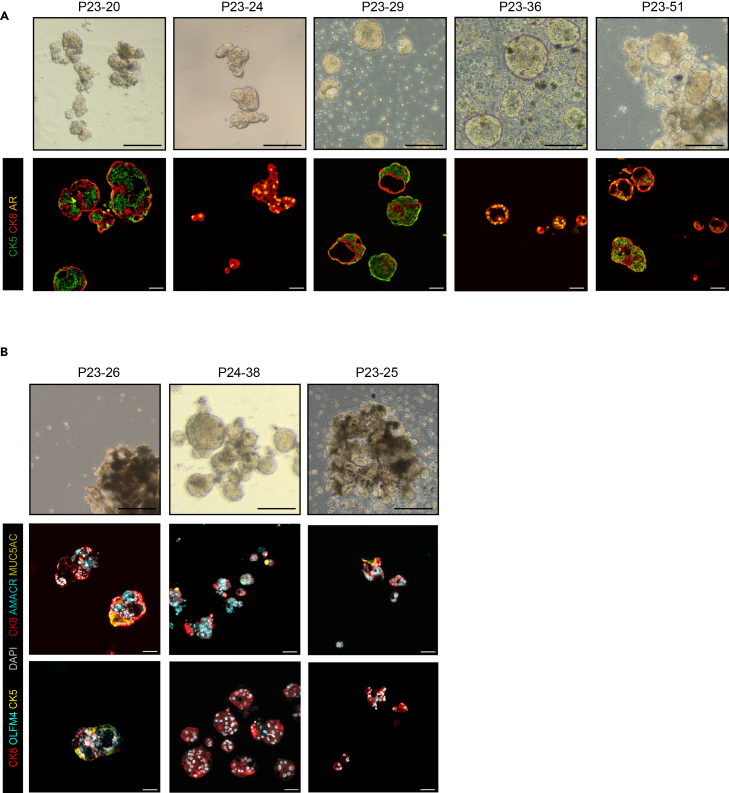
Organoid bright-field images and matching immunofluorescence images (A) Bright-field images of organoids (top) and representative immunofluorescence images of organoids stained with antibodies recognizing Cytokeratin 5 (CK5), Cytokeratin 8 (CK8), and the Androgen Receptor (AR) (bottom). Scale bars represent 200 μm for brightfield images and 50 μm for immunofluorescence images. Certain images are re-used from the associated Cell Reports publication. (B) Brightfield images of organoids (top) and representative immunofluorescence images of organoids stained with antibodies recognizing MUC5AC, AMACR, OLFM4, and CK5 (bottom). Scale bars represent 200 μm for brightfield images and 50 μm for immunofluorescence images. Certain images are re-used from the associated Cell Reports publication.

**Table 4:** Antibody dilution table

Antibody	Manufacturer	Reference	Dilution
CK5	Biolegend	905901	1:1000
CK8	Biolegend	904801	1:1000
AMACR	Cell Signaling	29256	1:500
OLFM4	Cell Signaling	14369	1:200
MUC5AC	Invitrogen	MA5-12178	1:100

***Note:*** Examples of matched brightfield and immunofluorescence images can be seen on Figures 6A and 6B.

***Note:*** Antibody dilutions for immunofluorescence analysis can be found in Table 4.

### Preparation of organoids for single-cell RNA sequencing

**Timing: 1–3 h**

39. Remove dead cells.
  a. Inspect the organoid culture plate under brightfield microscopy to confirm organoid formation.
  b. To avoid collecting single cells (i.e., cells which did not form organoids) that often remain at the bottom of the well, selectively transfer out organoids using a wide-bore pipette tip (e.g., P1000) to a new Falcon tube (Methods video S1).
  c. Inspect the organoid culture plate once again to ensure no organoids have been left behind.

***Note:*** If substantial groups of organoids are remaining, repeat the collection step.

40. Dissociate organoids using the same enzymatic protocol applied during organoid passaging.
  a. Following dissociation, transfer cells into ice-cold PBS–1% BSA and count the cells.

***Optional:*** If overall viability is <70%, perform Dead Cell Removal using a MACS-based annexin V kit.

***Note:*** A second round of depletion can further increase viability, albeit at the cost of reduced yield. In practice, sequencing runs have been successfully performed with viabilities as low as ∼60%, still producing satisfactory cell recoveries (>2,000 cells post-processing).

### Loading recommendations and platform notes

***Note:*** For subsequent scRNA-seq, we advice the readers to follow guidelines from 10× genomics and refer to the methods section of our publication.^1^ Furthermore, we highlight the following points:

***Note:*** Both chemistries have been validated. Notably, fixation required for the Flex protocol often results in cell recovery rates closer to the targeted loading. It has been hypothesized that certain fragile epithelial/tumor populations may lyse during microfluidic loading when processed fresh, potentially accounting for lower effective recoveries when using 3′ GEX protocol.

***Note:*** PCa PDOs often yield few cells after dissociation due to their limited expansion capacity, making it difficult to reach the input levels recommended by 10×. Nonetheless, for the Flex assay we proceeded with ∼50,000 cells instead of the suggested 300,000 and still obtained high-quality libraries and acceptable cell recovery.

41. Proceed to scRNA-seq using either 10× Genomics 3′ GEX (link) or 10× Genomics Flex (link) workflows following manufacturer recommendations.

### Prostate PDO single-cell atlas: Visualizing the atlas without coding

**Timing: 30 min**

***Note:*** To allow users exploring the data without coding skills, the Zenodo repository provides Loupe Browser files for each dataset. These files can be downloaded directly and opened locally using the free Loupe Browser application from 10× Genomics. The Loupe Browser software supports interactive visualization of metadata and gene-expression overlays on the original UMAP embeddings, but also allows user to create their own group of analysis based on custom criteria and perform differential gene expression.

**Figure 7:**
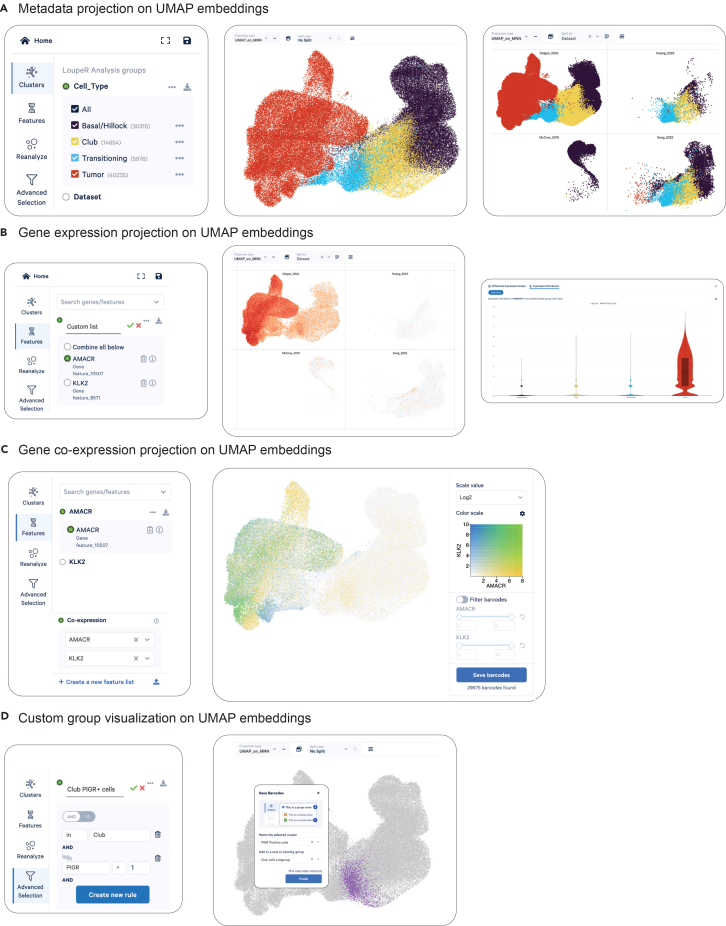
Metadata and gene expression visualization using Loupe Browser (A) UMAP embedding of the PPScA dataset colored according to the *Cell_Type* metadata. The left panel displays the Clusters tab, where users can select the metadata variable used for coloring. Right panels show the standard view and the split view (stratified by the *Dataset* metadata), illustrating how subgroup visualization can enhance interpretability. (B) UMAP embedding colored by the log2 expression of AMACR. The left panel displays the Features tab, where users can create and organize gene lists for expression queries. Right panels show the split view (according to *Dataset*) alongside a violin plot depicting AMACR expression across *Cell_Type* categories (selected in the Clusters tab). (C) UMAP embedding colored by the log2 co-expression of AMACR and KLK2. The left panel displays the Features tab, where users can set up a Co-expression list using previously defined feature list. (D) Custom group creation using logical operators to define a new metadata category. The left panel shows the set of filters applied in the Advanced Selection interface to identify PIGR-positive cells within the Club cell population. The right panels display the spatial distribution of the selected cells on the UMAP projection, along with the pop-up window used to finalize the creation of the new metadata group.

***Note:*** The upper toolbar allows users to enable a split view based on any metadata variable. This view preserves the original color scheme and displays separate UMAP panels for each subgroup, facilitating clearer stratification and comparative visualization across conditions or sample categories.

42. Download the desired.loupe file on the Zenodo repository.
  a. Go to: https://zenodo.org/uploads/17697351.
  b. Locate the dataset of interest and click “Download” next to the corresponding.loupe file (e.g., “Atlas_PCa_PDOs_loupe.cloupe).
43. Install Loupe Browser software.
  a. Go to: https://www.10xgenomics.com/support/software/loupe-browser/downloads.
  b. Follow instructions to download loupe browser.
44. Open the Loupe Browser file.
  a. Launch Loupe Browser.
  b. Either drag-and-drop the.loupe file onto the main window or double-click the file to open it directly.
45. Display metadata on UMAP embeddings (Figure 7A).
  a. Click the “Clusters” tab in the left panel.
  b. Select a metadata field (e.g., Cell_Type, Dataset, Culture_Condition) to color the UMAP accordingly.

46. Display gene expression on UMAP embeddings (Figure 7B).
  a. Click the “Features” tab in the left panel.
  b. Type the gene name into the search bar and press Enter.
  c. The UMAP will update to show expression levels for that gene.
  d. In the lower section, click Expression Distributions to display violin plot of the desired gene split by the metadata group chosen previously.

***Note:*** Expression is shown as Log2-transformed UMI counts. User can decide to display Log-Normalized to the total UMI count per cell by changing the scale value in the window located in the top-right corner.

***Note:*** Users may also visualize the combined expression of multiple genes. To do so, add additional genes to the list generated after entering the first gene, then enable “Combine all below”. Loupe Browser will compute a composite signal, and users can choose whether this combination reflects the maximum, minimum, average, or sum of the selected genes in the window located in the top-right corner.

47. Display co-expression patterns (Figure 7C).
  a. In the “Features” tab (left panel), click “+ Create a new feature list” at the bottom of the tab.
  b. Add the gene of interest to this list and name the list accordingly.
  c. Repeat the procedure to create a second single-gene list.
  d. In the lower section of the same tab, select “Co-expression”, then choose the two single-gene lists you created (e.g., *AMACR* and *KLK2*) to display their co-expression pattern on the UMAP plot.

***Note:*** Similarly, users may visualize the co-expression of a list containing multiple genes. When selecting more than one gene in the co-expression view, Loupe Browser will prompt the user to choose how the combined signal should be computed—using the maximum, minimum, average, or sum of the selected genes.

48. Create custom analysis groups (Figure 7D).
  a. In the upper toolbar, select the lasso tool and choose either “Freehand” or “Rectangle” selection.
  b. Draw the selection directly on the UMAP plot (either in split view or standard view).
  c. In the pop-up window, assign a name to the newly created cluster and a name to the corresponding metadata category; this allows additional clusters to be grouped under the same category if needed.
  d. The new group and cluster will now appear in the Clusters tab and can be analyzed in the same way as any other metadata group.

***Note:*** Using the same workflow, users may also create clusters based on expression patterns by using the “Advanced Selection” tab in the left panel. This interface allows the definition of logical rules (e.g., AND, OR, ≥, ≤, ≠) to select cells according to specific gene-expression thresholds or combinations, enabling more targeted and rule-based subgroup creation.

### Recreating all data objects independently (coding required)

**Timing: 30 min**

***Note:*** For user who might want use the processed directly into R, both the Zenodo and GEO repositories also provide standardized Matrix Exchange (MEX) files (matrix.mtx, features.tsv, barcodes.tsv, and an accompanying metadata.csv) allowing users to reconstruct the processed objects with minimal scripting. Upon reconstruction (e.g., using Seurat or Bioconductor frameworks, the resulting objects are pre-populated with the original dimensionality reduction (UMAP) and annotations, including UMAP coordinates, normalization factors, clustering, cell type assignments and cell-cycle scores.

49. Download set of MEX files.
  a. Create a local folder on your computer named after the group of samples you are planing to analyse (e.g., “Atlas_PCa_PDOs”, “SR10_sample_1”).
  b. Go to the Zenodo or GEO repository (GSE305752) and download the set of files corresponding to your whishes (e.g., “Atlas_PCa_PDOs_matrix.mtx”, “Atlas_PCa_PDOs_features.tsv”, “Atlas_PCa_PDOs_barcodes.tsv”, “Atlas_PCa_PDOs_metadata.csv”).

***Note:*** In the GEO repository, the file GSE305752_README.txt provides a detailed explanation of the file nomenclature. In the Zenodo repository, the same information is presented in the “Additional Description” table accompanying each file.

50. Load necessary packages and set directory.

# Core packages

library(Matrix)  # readMM for MEX

library(SingleCellExperiment) # for SCE

library(Seurat)  # for Seurat objects

data_dir <- “path/to/MEX_folder” # change this

51. Read MEX files.

# Load counts matrix (sparse)

counts <- readMM(file.path(data_dir, "matrix.mtx"))

# Load features (genes)

features <- read.table(

 file.path(data_dir, “features.tsv”),

 sep = “\t”,

 header = FALSE,

 stringsAsFactors = FALSE)

# Load barcodes (cells)

barcodes <- read.table(

 file.path(data_dir, “barcodes.tsv”),

 sep = “\t”,

 header = FALSE,

 stringsAsFactors = FALSE)

# Optional: per-cell metadata

metadata <- read.csv(

 file.path(data_dir, “metadata.csv”),

 row.names = 1,

 stringsAsFactors = FALSE)

52. Build a SingleCellExperiment or Seurat object.

# Build SingleCellExperiment object

sce_obj <- SingleCellExperiment(

 assays = list(counts = counts),

 colData = metadata)

# Build Seurat object

seurat_obj <- CreateSeuratObject(

 counts = counts,

 meta.data = metadata)

***Note:*** All code used to generate the processed datasets, intermediate objects, and figures reported in the accompanying study is available in the associated GitHub repository. The repository includes the complete computational workflow, including scripts for data ingestion, quality control, harmonization, integration, downstream analyses, and final export. Input files consist of (i) count matrices generated in this study and (ii) publicly released datasets from previously published work. A detailed **README** file describes the repository structure and provides instructions for reproducing each dataset.

## Expected outcomes

The primary outcome of this protocol is the generation of PCa PDOs derived from all clinical sample types. These PCa PDOs vary in cell composition, and therefore their in-depth characterization is essential to their use in translational research. A secondary outcome is the single-cell transcriptomic profiling of PDOs and the utilization of the Prostate PDO single-cell Atlas (PPScA).

## Limitations

The two main limitations of this protocol are the short-term growth of the PCa PDOs, and the heterogenous cell-type composition. We were able to maintain these PCa PDOs for a maximum of 5 passages, as the cells do not amplify enough to counterbalance the cells lost during the passaging process. The cell-type composition as well as the take-rate of the PCa PDOs is highly patient and sample-dependent, making the sample selection and characterization steps essential.

## Troubleshooting

### Problem 1

Large pieces of tissue remain after maximum recommended tissue dissociation time (5 × 10 min).

### Potential solution

**Figure 8:**
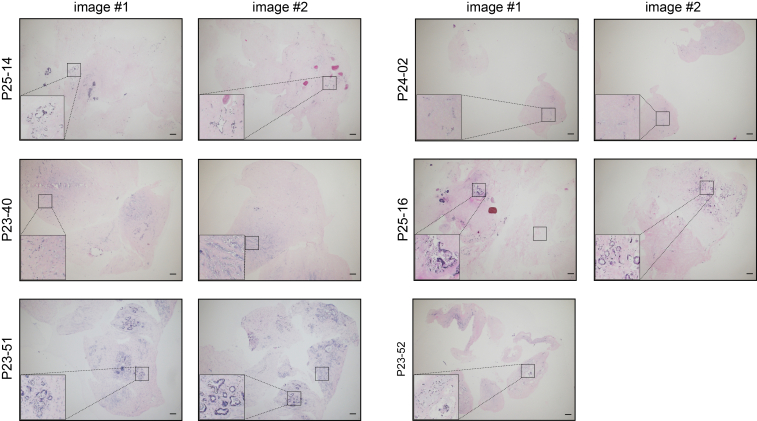
H&E images of tissues post-dissociation Scale bars represent 20 μm.

In our experience, and as shown in Figure 8, the pieces remaining after tissue digestion (3 × 10 min) are stromal-rich and acellular regions with very few epithelial cells remaining and can therefore be discarded.

### Problem 2

Organoids appear larger than normal and black, or you obtain low viability after passaging.

### Potential solution

**Figure 9:**
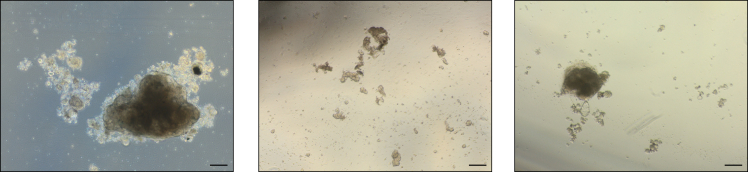
Brightfield images highlighting organoids in need of passaging Scale bars represent 200 μm.

If the organoids have an appearance similar to Figure 9, they should be passaged. For some organoid cultures, passages should be performed as often as every 5 days. If the organoids get too large, the center will become necrotic, and cells will eventually be lost.

### Problem 3

Organoids do not form.

### Potential solution

**Figure 10:**
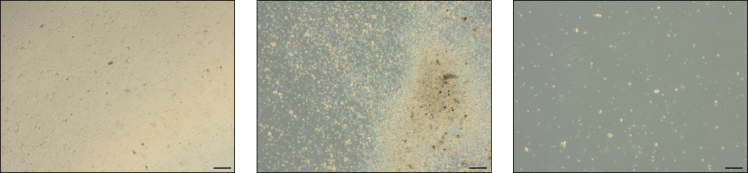
Unsuccessful organoid growth Scale bars represent 200 μm.

If organoids do not form after 7 days, as shown in Figure 10, the sample should be discarded and considered as a failure.

## Resource availability

### Lead contact

Requests for further information and resources should be directed to and will be fulfilled by the lead contact, Clémentine Le Magnen (clementine.lemagnen@usb.ch).

### Technical contact

Technical questions on executing this protocol should be directed to and will be answered by the technical contact, Clémentine Le Magnen (clementine.lemagnen@usb.ch).

### Materials availability

This study did not generate new unique reagents.

### Data and code availability

• Raw single-cell RNA sequencing data generated in this study are published on EGA (EGA study: EGAS50000000807). The two datasets can be found with the following IDs: EGAD50000001185 (GEX 3′ scRNA-seq) and EGAD50000001186 (Flex scRNA-seq).
• Pre-processed data for all single-cell datasets generated in this study, together with reanalyzed datasets from previous publications and various integrations (e.g., PPScA), are publicly available through the Gene Expression Omnibus repository (GEO: GSE305752) and Zenodo (https://doi.org/10.5281/zenodo.17697351).
• Code for processing and analysis is released on GitHub (https://github.com/LeMagnenLab/Prostate_PDOs_single_cell_Atlas).

## Acknowledgments

We thank all patients who consented to participate in the study and all clinicians, pathologists, and study nurses involved in sample acquisition. We thank the Next Generation Sequencing Platform of the University of Bern for performing the scRNA-seq. Financial support was provided by funding from 10.13039/501100006069Krebsliga beider Basel (KLbB-5329-03-2021 to C.L.M.), the 10.13039/501100001711Swiss National Science Foundation (320030_205086 to C.L.M.), the 10.13039/501100013362Swiss Cancer Research Foundation (KFS-5552-02-2022 to A.M., C.L.M., and C.A.R.), the 10.13039/100008375University of Basel (U.330.1033 to R.P.), and the Department of Surgery of the 10.13039/100016015University Hospital Basel (PMC Platform to C.L.M.). Components of several figures were created with BioRender.com.

## Author contributions

R.D., C.L.M., and R.P. conceived the study. C.L.M. supervised the study. R.D., R.P., and J.W. designed and conducted the experiments. R.D. and R.P. analyzed the data and performed data visualization. A.J.T., K.D.M., H.P., H.S., A.M., T.V., C.A.R., and L.B. selected and enrolled patients in the study and contributed to the collection of samples, clinical data, and pathological data. T.V. and L.B. performed the pathological evaluation and designed Figure 1. R.D., C.L.M., and R.P. wrote the initial manuscript. All the authors contributed to the final version.

## Declaration of interests

The authors declare no competing interests.

## Supplemental information

Methods video S1. Collection of organoids using a wide-bore pipette tip, related to step 39 Organoids are collected with a wide-bore pipette tip to leave behind single-cells and dead cells.
